# Wearable multichannel pulse condition monitoring system based on flexible pressure sensor arrays

**DOI:** 10.1038/s41378-022-00349-3

**Published:** 2022-02-08

**Authors:** Jie Wang, Yirun Zhu, Zhiyong Wu, Yunlin Zhang, Jian Lin, Tao Chen, Huicong Liu, Fengxia Wang, Lining Sun

**Affiliations:** 1 Jiangsu Provincial Key Laboratory of Advanced Robotics, School of Mechanical and Electric Engineering, Soochow University, Suzhou, 215123 China; 2 Micro Nano System Research Center, Key Laboratory of Advanced Transducers and Intelligent Control System of Ministry of Education and Shanxi Province & College of Information Engineering, Taiyuan University of Technology, Taiyuan, 030024 China; 3 Suzhou Institute of Nano-tech and Nano-bionics, Chinese Academy of Sciences, Suzhou, 215123 China

**Keywords:** Electronic devices, Sensors, Electrical and electronic engineering

## Abstract

Pulse diagnosis is an irreplaceable part of traditional Chinese medical science. However, application of the traditional pulse monitoring method was restricted in the modernization of Chinese medical science since it was difficult to capture real signals and integrate obscure feelings with a modern data platform. Herein, a novel multichannel pulse monitoring platform based on traditional Chinese medical science pulse theory and wearable electronics was proposed. The pulse sensing platform simultaneously detected pulse conditions at three pulse positions (Chi, Cun, and Guan). These signals were fitted to smooth surfaces to enable 3-dimensional pulse mapping, which vividly revealed the shape of the pulse length and width and compensated for the shortcomings of traditional single-point pulse sensors. Moreover, the pulse sensing system could measure the pulse signals from different individuals with different conditions and distinguish the differences in pulse signals. In addition, this system could provide full information on the temporal and spatial dimensions of a person’s pulse waveform, which is similar to the true feelings of doctors’ fingertips. This innovative, cost-effective, easily designed pulse monitoring platform based on flexible pressure sensor arrays may provide novel applications in modernization of Chinese medical science or intelligent health care.

## Introduction

As the essence of China, the effectiveness and uniqueness of traditional Chinese medical science (TCMS) in clinical diagnosis has been well verified for 2000 years^1,2^. Pulse diagnosis, one of four TCMS diagnostic methods, has already shown unique advantages in long-term clinical practice^3^. However, TCMS physicians have no clear uniform standard for judging pulse conditions during clinical treatment, and the accuracy of their diagnosis is limited by the subjective feelings and experience of doctors^4,5^. In addition, the traditional pulse diagnostic method has difficulty integrating obscure feelings with modern data platforms. Thus, there are still great challenges for application of TCMS in modern or intelligent health care ^6,7^. In general, detecting pulse signals and extracting obscure concepts, such as position, number, shape, and trend, and presenting them specifically and parametrically was the key factor in promoting TCMS modernization.

With the development of modern TCMS and intelligent health care, researchers in different fields have expended more effort to obtain pulse condition information^8–10^. Sensors using different detection mechanisms, such as optics, image processing, acoustics, and pressure, etc., were all used previously to detect human pulse waves. Currently, wearable pressure sensors are proposed for monitoring arterial pulse conduction since pulse detection, in essence, captures multidimensional signals under a loading force^11–13^. Characteristic points of pulse waveforms, such as percussion waves (P-waves), tidal waves (T-waves), and diastolic waves (D-waves), can be detected due to their high sensitivities^14–16^. However, most sensing platforms for pulse signals are based on single point sensors due to their large sizes and rigid structures^17–24^. Great challenges still apply when integrating large sensors into an array designed to detect pressure signals within a small area^25–33^. Therefore, reported sensors still have limited applications in wearable pulse diagnosis systems.

According to the three positions and nine indicators TCMS theory, there is great value in investigating pulse length, width and strength distribution, since this could reveal pulse conditions^34^. Chu et al. purposed using sensor arrays to analyze different pulse conditions^35^. They achieved great progress in developing pulse monitoring systems since their system showed pulses at three positions simultaneously. However, the system could not provide temporal and spatial dimensions of pulse conditions like those of a doctor’s examination, because there was only one sensor at each position. In particular, few studies have considered spatial distributions among the three pulse monitoring positions, even though pulse spatial distribution is important information for a doctor’s diagnosis in TCMS^36–38^. This was mainly attributed to the lack of an available method for detecting multidimensional signals and analyzing weak pulse signals^39–41^.

In this work, a novel multichannel pulse monitoring platform based on TCMS pulse theory and flexible pressure sensor arrays was proposed. The obtained platform was flexible and wearable, and it is comparable to the system based on a rigid sensor. Moreover, it simultaneously obtained 3-dimensional pulse signals at three pulse positions (Chi, Cun, and Guan), which exceeded the performance of systems reported in the literature^41–43^. Pulse signals from different individuals with different conditions were collected and analyzed. Moreover, the spatial distribution of the three-position pulse signals was also investigated, which, like the signals mentioned above, allowed deeper consideration of subtle human characteristics and different pulse conditions and revealed different pathological features. In addition, all temporal and spatial information for a person’s pulse waveform was analyzed by incorporating the data from the pressure sensor arrays into a 3-D map, which was similar to the sensations determined with doctors’ fingertips. The proposed multichannel pulse monitoring system for TCMS diagnosis and a novel analysis approach for palpation are important in promoting the development of modern Chinese medicine science.

## Results and discussion

### Pulse sensing platform

**Fig. 1:**
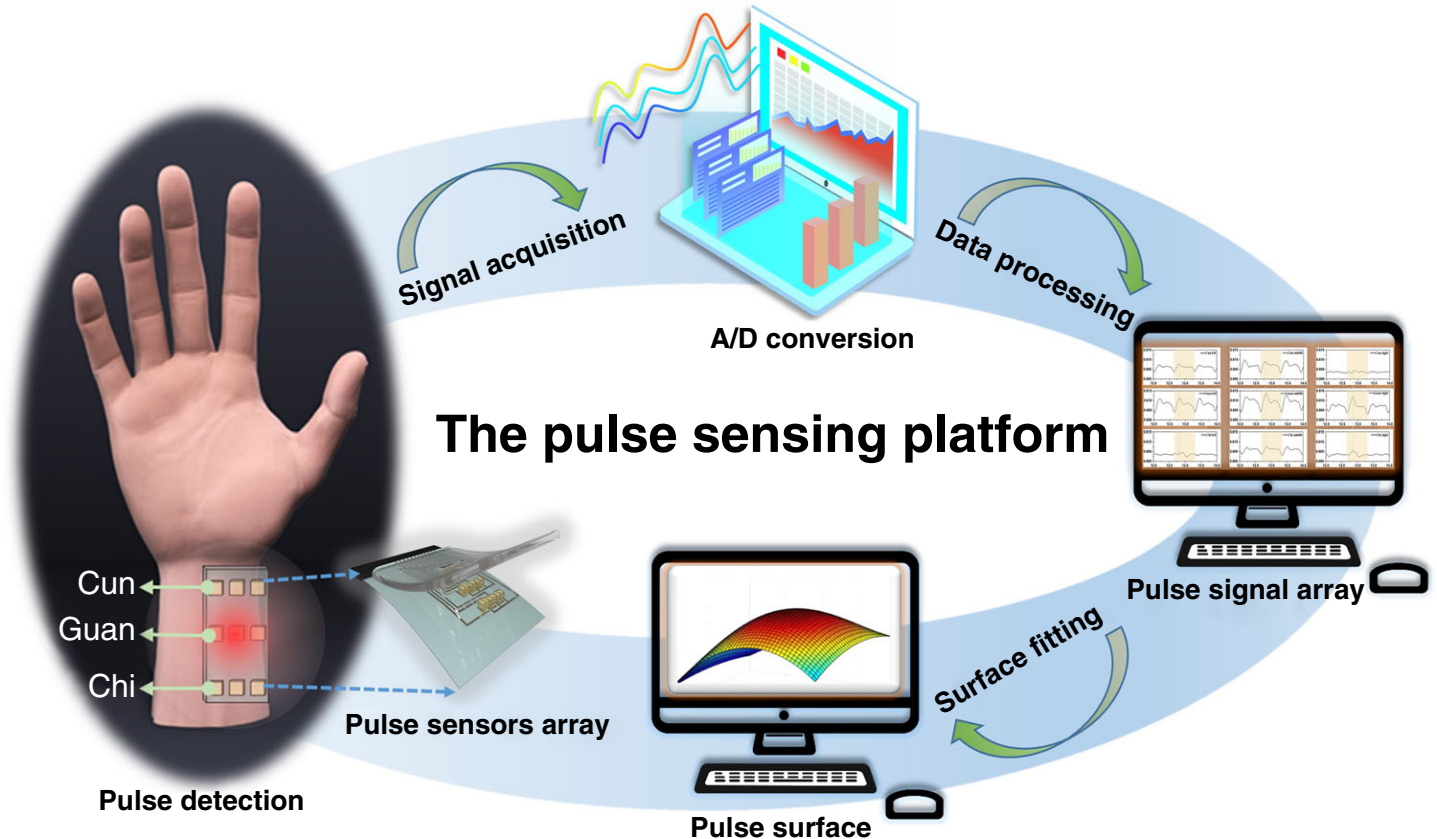
Schematic of the pulse sensing platform. The sensing platform consists of sensor arrays, signals processing, signals displaying and three-dimensional signals fitting

A schematic of the pulse sensing platform is shown in Fig. 1. The pulse sensing platform system consisted of flexible pressure sensor arrays and signal acquisition, processing, and fitting equipment. In this case, 3 × 3 pressure sensor arrays were distributed at the Cun, Guan, and Chi pulse locations to detect pulse signals while following TCMS practice (Fig. S1). With the cardiac ejection of blood, the arteries at these locations experience wave fluctuations resulting in different patterns corresponding to blood volume, pressure and flow. Signal acquisition and processing mainly included conversion, amplification, denoising, and detection of electric signals. The signal fitting process was used to fit smooth surfaces and form a 3-dimensional pulse map. Thus, the sensing platform based on pressure sensor arrays could simultaneously capture pulse signals from different locations. The system also showed multidimensional pulses from each location. Moreover, together with the signal fitting process, the sensing platform showed three-dimensional maps of the pulse and revealed the shapes of pulse lengths and widths, which was similar to the information gathered by a doctor.

**Fig. 2:**
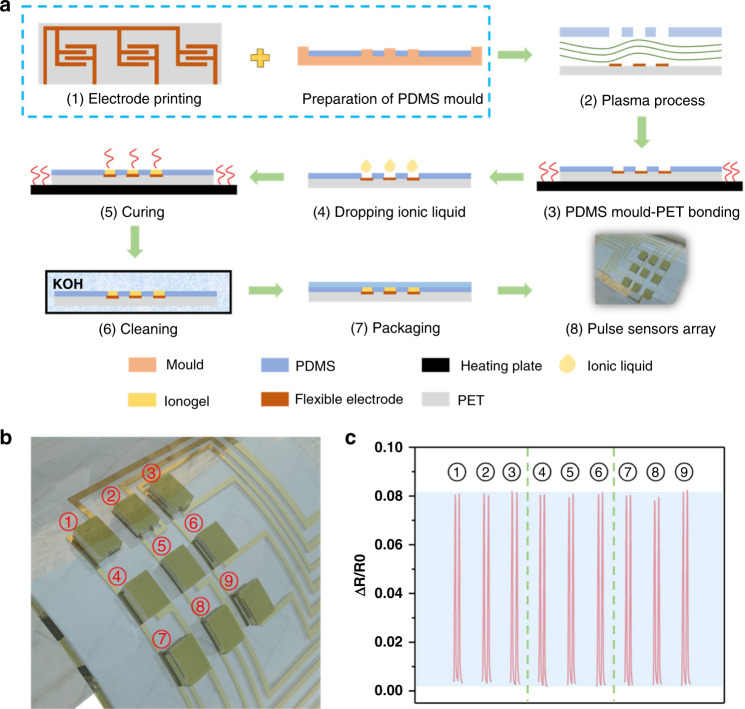
Sensor preparation flow chart, array diagram, and signal response. **a** Schematic of fabrication details for the pressure sensor array. **b** Nine sensor elements of the obtained pressure sensor array. **c** Resistance change rate response of nine sensor elements subjected to the same pressure

Due to their excellent flexibility and high sensitivity, ionogel-based 3 × 3 pressure sensor arrays were used in this sensing platform^44,45^. According to simulations of ionogels, sensing the dynamic and static pressures of pulses could be difficult (Fig. S2). The structure of the sensor array is shown in Fig. S3. The process used for fabrication of the sensing system is shown in Fig. 2a, and a detailed description is given in the Experimental section. In brief, Ag lines serving as electrodes were printed on a flexible PET substrate by the screening printing method. Then, arrayed ionogel films were deposited on the surface of the Ag electrodes with patterned PDMS. Finally, the PDMS film was used to improve stability. The uniformity of pressure sensor arrays was investigated and is shown in Fig. 2b, c. All sensor elements exhibited similar change ranges when subjected to the same external pressure force. These results indicated that the pressure sensor arrays had great consistency and stable pressure sensing performance, which ensured their feasibility for pulse detection.

### Two-dimensional pulse signals

**Fig. 3:**
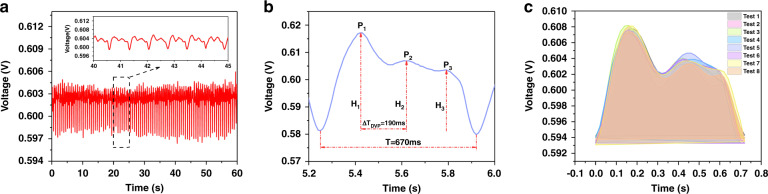
Waveforms and parameters for pulses and stability of the sensor. **a** Pulse waveform recorded for a volunteer over 60 s. **b** Complete pulse waveform for one cardiac cycle. Different parameters used to calculate health information were also studied, including stiffness of the vasculature and blood flow velocity. **c** Stabilities of pulse sensors

A continuous series of pulse-taking experiments was conducted. First, the pulse waveform was measured and is shown in Fig. 3a. The shapes of all pulse cycles were similar and showed response times of ~670 ms. Moreover, the obtained pulse waveforms included forward waves, reflected waves, and three peaks (Fig. 3b), which were consistent with TCMS theory. As a result of ventricular contraction, the early systolic peak (P_1_) resulted when blood was squeezed out of the heart; this was followed by an inflection point (P_2_). During aortic valve closure, the dicrotic notch and dicrotic peak (P_3_) resulted when blood flowed from the heart. In addition, pulse waveforms were recorded by different sensor arrays at the same position and are shown in Fig. 3c. The shapes of the pulse cycles were similar, and little baseline fluctuation was observed; this demonstrated that the sensor arrays effectively recorded the changes in pulse waveforms. It also showed that the obtained sensors were consistent and stable, which are vital for detecting multidimensional signals.

**Fig. 4:**
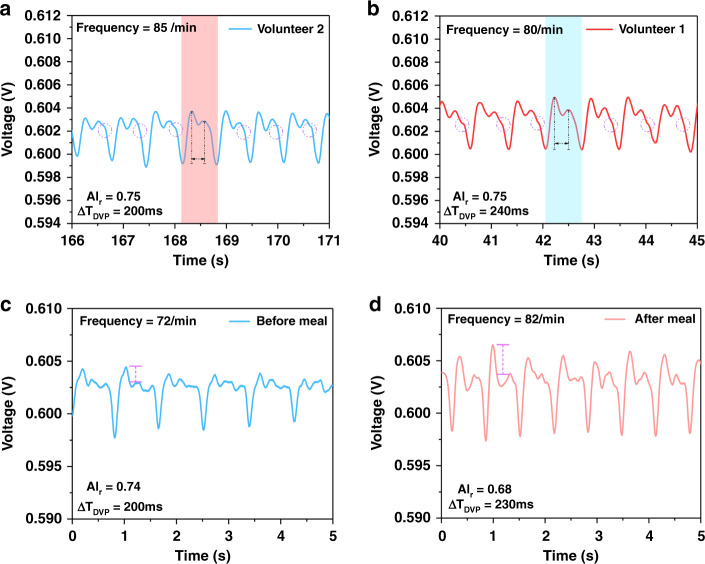
Pulse waveforms over 5s with significantly different characteristics for different volunteers with different circumstances. **a** Pulse waveform showed gentle features. **b** Pulse waveform with a more obvious dicrotic notch. **c** Stable and gentle pulse waveform taken before a meal. **d** Pulse waveform with higher early systolic wave value, taken after a meal

Pulse signals from different individuals and conditions were measured by this pulse sensing platform, and the results are shown in Fig. 4. The pulse waveforms of the different volunteers were basically the same except for small changes in frequency. Volunteer 1 and Volunteer 2 showed pulse frequencies of 85/min and 80/min (insets of Fig. 4a and b), respectively, which were similar to those reported in the literature for healthy adults^35^. Finally, the pulse wave showed a steady downward trend during relaxation, which is the diastolic wave. All results demonstrated that the platform clearly detected the two-dimensional pulse signals.

The time domain analysis method is based on differential equations used to determine the time response of the system directly. The performance of the system is analyzed based on expressions for the response and time response. Herein, a time domain analysis method was first used to study the meaning of the pulse waveform in TCMS pulse theory (Table S1)^46,47^. Different parameters extracted from the pulse waveform could reflect different physiological conditions (Table S2)^46,47^. To reflect the characteristics of the pulse wave more accurately, more common construction parameters were calculated based on the above time domain parameters (Table S3). The time interval between the systolic peak and dicrotic peak was defined as the digital volume pulse (ΔT_DVP_=${\text{T}}_{{\text{P}}_{2}}$−${\text{T}}_{{\text{P}}_{1}}$), and the radial artery augmentation index, defined as AI_r_ = P_2_/P_1_, is highly related to the physical condition of the body. According to TCMS, healthy and elastic arteries usually exhibit small AI_r_ and long ΔT_DVP_. The inflection point and dicrotic peak tend to disappear from the pulse waveforms of older people; this is due to high stiffness and poor elasticity of blood vessels, which lead to high AI_r_ and short ΔT_DVP_. As shown in Table S4, the ΔT_DVP_ values for Volunteer 1 and Volunteer 2 were 210 ms and 230 ms, respectively, and their AI_r_ values were 0.74 and 0.8, respectively. These results showed that the volunteers had long ΔT_DVP_ and small AI_r_, further indicating that they were healthy. Similar pulses were obtained from different volunteers, which confirmed that the parameters for the two volunteers’ pulses were obviously different. The ΔT_DVP_ for Volunteer 2 was longer than that for Volunteer 1. Moreover, after 10 acquisition experiments and averaging of the parameters (Table S4), the latter had a clearer dicrotic peak than the former, demonstrating different cardiac ejection functions for the two volunteers. The pulse conditions for healthy youths constituted normal pulses according to the parameter K^47^. Clearly, the proposed pulse sensing platform provided valuable information for evaluating physical condition, which is important in demonstrating applications in intelligent health care.

More importantly, the pulse sensing system based on pressure sensor arrays distinguished differences in the physical conditions of volunteers before and after meals. The pulse waveforms of the volunteers showed steady trends before a meal, while the systolic peak became much higher after a meal. These results indicated that postprandial blood flow was strong. As shown in Table S5^15,46,47^, the pulse signal measured before a meal showed a lower AI_r_ value and higher frequency than that measured after a meal. This was because the human body exhibited faster blood flow toward the stomach for digestion, resulting in higher heartbeat frequency and stronger ejection force. The pulse condition in the satiated state was identified as a slippery pulse according to parameter K. The parameter K is the ratio of the two parameters

$$\left(\frac{{\mathrm{P}}_{\mathrm{m}}-{\mathrm{P}}_{\mathrm{d}}}{{{\mathrm{P}}}_{{\mathrm{H1}}}-{\mathrm{P}}_{\mathrm{d}}}\right)$$

where P_m_ is the average pulse, and P_H1_ and P_d_ represent the peak and trough of the pulse wave, respectively.

### Three-dimensional pulse signals

**Fig. 5:**
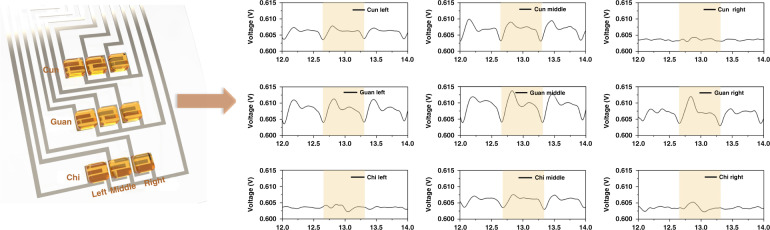
Summary for each channel that corresponds to pulse pressure signals taken from nine sensing elements on the wrist. The positions of the nine sensor arrays detecting the pulse signals (Cun, Guan, Chi) shown on the left; Corresponding pulse signals (Cun, Guan, Chi) shown on the right

In this work, the acquisition system simultaneously collected voltage signals with 9-channel sensor arrays located at the Cun, Guan, and Chi positions. Thus, the sensing platform could sense pulse signals in 2 dimensions, as reported in the literature^48–50^, but also provided multidimensional signals that were similar to those from TCMS doctors^47^. The nine sensing elements were located on the wrist plane, and each element contacted the skin surface effectively due to the high flexibility of the sensor arrays. The pulse waveforms for each position are shown in Fig. 5, which indicates that nine channels operated normally and detected pulse signals. Moreover, the peaks and valleys of the waveforms corresponded to each other, which confirmed good synchronization for this multichannel acquisition system. Each channel illustrated the relationship between pulse strength and time.

**Fig. 6:**
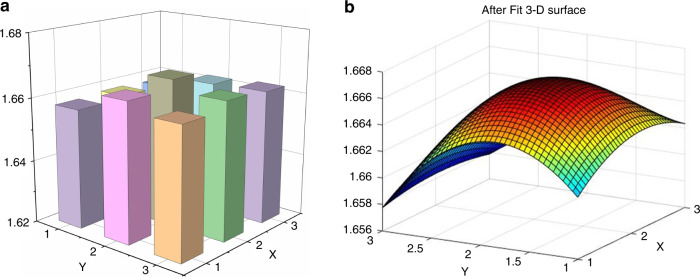
Intensity histogram and signal fitting diagram for pulse. **a** Column diagrams for pulse strength distribution. **b** A 3-dimensional surface of the radial artery pulse was constructed from nine-channel pulse signals through the polynomial fitting method. The strength distribution, pulse length, pulse width and shape were clearly characterized

To investigate the temporal and spatial dimensions of the pulse wave, it was important to consider pulse strength mapping in spatial dimensions for each channel. A column diagram composed of nine points is shown in Fig. 6a, where each point represents pulse intensity at the wrist. The *X* axis corresponded to pulse width, and the *Y* axis represented pulse length and different positions. The *Z* axis shows the pulse amplitude of each position. The column diagram for pulse strength distribution describes pulse changes at the Cun, Guan, and Chi positions. To explain the changes in the pulse signals more clearly, a smooth 3-dimensional pulse mapping surface was used to simulate the fingertip sensations of a TCMS physician; discrete columns were too abstract to determine the pulse current and strength distribution. The surface fitting method was used to construct a smooth and coherent surface with enough points to show strength trends. The real-time display of the 3-dimensional surface was realized in LabVIEW with cubic spline interpolation, which presented dynamic changes in the pulse shape in real time during pulse monitoring. The surface fitting process was coded based on MATLAB. A cubic fitting method was used to connect the signals from each sensing element and construct a pulse strength mapping surface. As shown in Fig. 6b, the pulse strength along the radial artery was stronger than those in the surrounding area, and position Guan exhibited a greater pulse strength than positions Cun and Chi, which was consistent with literature reports.

**Fig. 7:**
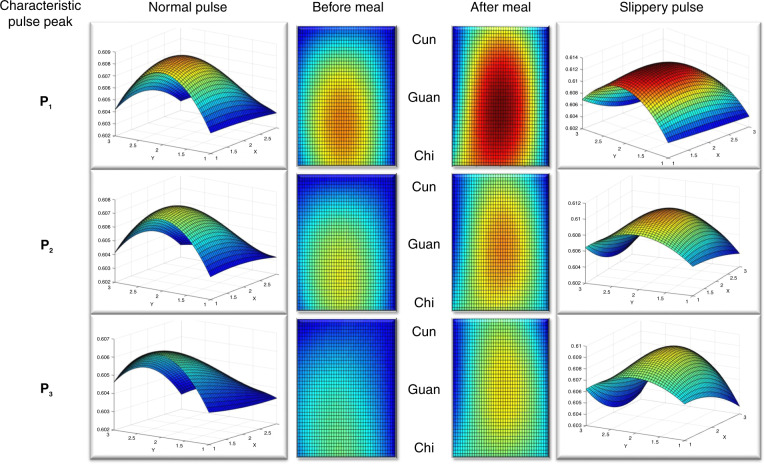
Strength distributions of 3D surfaces corresponding with three characteristic peaks on pulse waveforms taken under different pulse conditions over time. The left of the figure is the pulse signal before meal, and the right is the pulse signal after meal. Apparently, the pulse beats stronger after meal

Strength distributions for complete pulse cycles under different physiological conditions were also studied. According to TCMS theory, the health conditions of different human organs are closely related to pulse shapes at different positions on the radial artery (the Cun, Guan, and Chi positions). Figure 7 shows front and overhead views of a 3-D surface revealing the strength distribution and flow trend for pulse signals in spatial dimensions. The *X* axis and *Y* axis represent pulse width and length, respectively. The three pictures corresponded to the three sticking points of the pulse cycle under different conditions. Differences before/after a meal were easily determined because the overall pulse strength was significantly greater after a meal. The stomach had stronger activity after dinner, and the rate of blood flow also increased. Importantly, according to TCMS theory^15,51^, the pulse position corresponding to the stomach was exactly Guan. Thus, the pulse was also longer and wider, which was consistent with the greater strength of the pulse.

**Fig. 8:**
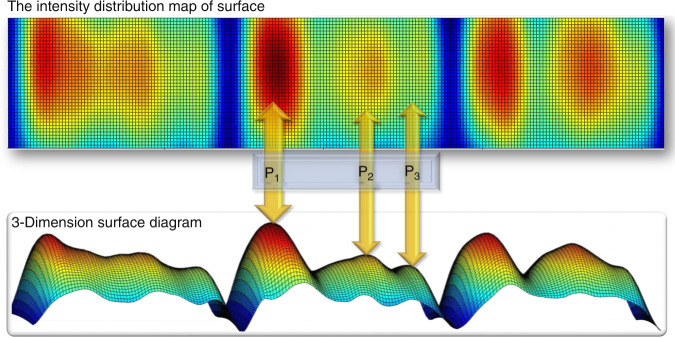
3-dimensional surface diagram for the pulse waveform; the time axis shows the intact pulse shape for every pulse cycle, which represents the sensations of a doctor’s fingertips at a specific pulse position. The intensity distribution map revealed the intensity and width of the pulse at each characteristic peak. P_1_, P_2_, and P_3_ represent the systolic peak, the point of inflection and the point of the dicrotic peak, respectively

Blood pressure was also regarded as a flow surface composed of pulse pressures recorded from the Cun, Guan, and Chi positions. The temporal pulse strength distribution was investigated first and is shown in Fig. 8. The curves revealed the overall pulse flow trends for every pulse cycle and presented abstract pulse shapes. The shape of the resulting fitting surface reflected the intensity of each pulse cycle, and the overall shape trend was superior to that reported in the literature^51^. At the same time, the color of the surface reflected the intensities of the pulse at corresponding positions on the human wrist. In the future, more physical information may be found by analyzing pulse shapes more intensively. In summary, this work provided a novel way to detect pulses condition and analyze pulse signals, which benefits the development of TCMS.

## Conclusions

This paper reports a novel multichannel pulse sensing platform based on TCMS pulse theory and a wearable pressure sensor array. The pulse sensing system was flexible and wearable. Importantly, it accurately detected and recorded pulse signals at three pulse positions (Chi, Cun, and Guan) simultaneously. These signals were fitted to smooth surfaces to form 3-dimensional pulse maps, which revealed the shapes of pulse length and width vividly; this could compensate for the shortcomings of traditional single-point pulse sensors. Moreover, the pulse sensing system based on pressure sensor arrays accurately measured pulse signals for different individuals with different conditions and distinguished the differences caused by physical conditions of volunteers. In addition, this system provided full temporal and spatial information for pulse waveforms, which was similar to the feelings of doctors’ fingertips. This innovative pulse sensing platform may stimulate new developments for modernization of intelligent TCMS.

## Experimental section

### Pulse condition sensing system

The pulse sensing system was composed of pressure sensor arrays, a preprocessing circuit and a multichannel data acquisition platform. The pressure sensor arrays converted pulse changes into resistance changes that were sent to the data acquisition system. A preprocessing circuit was designed to turn the resistance signals into voltage signals since voltage was much easier to collect with the virtual instrument. In addition, this preprocessing circuit also included a multistage amplification module and a low-pass filter. The amplification module was used to amplify the magnitude of the pulse signal, and the filter was designed to prevent high-frequency noise, which made the pulse signal smoother. As a result, it was easier to distinguish characteristic peaks in the pulse wave information, and it was an important foundation allowing researchers to judge the pulse condition. The multichannel pulse signals were transferred into an acquisition system in the form of voltage variations. The acquisition system was based on a data acquisition card (NI DAQ Card USB-6343), which realized analog-to-digital conversion. The data acquisition process was controlled by LabVIEW. The sampling frequency of each channel was 50–100 Hz, which effectively recorded the characteristic shapes of pulse waves since the pulse frequency was usually below 20 Hz. The acquisition program accepted multichannel voltage data and sorted them an array for output. The multichannel signals were used to construct a 3-dimensional map with the polynomial fitting equation in LabVIEW. The refresh frequency of the fitting surface was set to 100 Hz to show 3-D mapping of pulse conditions in real time.

### Layout design for the pressure sensor arrays

#### Sensor array sizes

According to the book “Shang Han Lun” on TCMS, the radial artery pulse range is approximately 9 mm in width and 3 cm in length. Thus, we designed 3 × 3 pressure sensor arrays to detect the widths and mapping information for pulses at Cun, Guan, and Chi. Each position had three pressure sensors, every sensing element measured 2 mm × 2 mm, and the thickness was approximately 1 mm. The dimensions of sensing arrays with 3×3 units were 3 cm in length and 0.9 cm in width. The elements of the sensor arrays were arranged in rows and columns; rows were defined as the *Y* axis and columns were defined as the X axis. The distance between each row on the *Y* axis was 10 mm, and the distance between each column on the *X* axis was 1.5 mm. The three rows were used to detect the pulse signals at different positions of the radial artery (Cun, Guan, and Chi), and the lengths of the pulses were also revealed. The lengths of pulses mimicked examinations with three fingers (index finger, middle finger, and ring finger) in TCMS. In addition, the three columns were used to detect pulse strength. Each sensing element was numbered and connected to the acquisition system sequentially, and this corresponded with the 3-dimensional distribution coordinates shown in our monitor.

#### Fabrication process for pressure arrays

The brief fabrication process is described as follows: first, the flexible and transparent substrate was made inside a 3-D pattern with polydimethylsiloxane (PDMS). The flexible electrode was screen printed on 25 μm thick PET with conductive silver paste (HFSP-53). The patterned PDMS and electrode layers were plasma processed and connected immediately in a clean environment to bond their surfaces firmly, which guaranteed tight connections between PDMS and the electrode layer without any gaps. Nine ionogel elements with thicknesses of 1 mm, widths of 2 mm, and lengths of 2 mm were used as sensing layers detecting pulse conditions, and their fabrication process was similar to that reported in the literature.^52^ Finally, the ionogel layers were packaged with Eco-flex to avoid external contact, which might affect their sensing performance.

#### Characteristic features of pulses

Pulse intensities were sensed and recorded with the flexible pulse sensor arrays and pulse condition measuring system mentioned previously (Fig. S4). Deformations of the ionogel-based sensing elements converted pulse changes into resistance changes, which were sent to the measuring system for fitting, storage, and real-time presentation. Volunteers were asked to sit still for 15 m before the experiment and not to move the arm that would be palpated next to keep blood flow in a normal state. Then, the designed pulse sensor arrays were attached to the wrist according to the three pulse positions. During the experiment, external pressure was applied to the wrist with three fingers through the sensor arrays, and then pulse signals were recorded and stored by the system.

## Supplementary information

Supplementary informantion
