# Supplementary material for: Wearable multichannel pulse condition monitoring system based on flexible pressure sensor arrays
Source: Microsyst Nanoeng. 2022 Feb 8;8:16. doi: 10.1038/s41378-022-00349-3 (PMC8821641; doi:10.1038/s41378-022-00349-3)
Supplement: Supplementary file 1 — Supplementary informantion [file 41378_2022_349_MOESM1_ESM.docx]

Supplementary Information

**Wearable multi-channels Pulse Condition Monitoring System Based on Flexible Pressure Sensors Arrays**

Jie Wang^1,2#^, Yirun Zhu^1#^, Zhiyong Wu^1^, Yunlin Zhang^1^, Jian Lin^3^*, Tao Chen ^1^, Huicong Liu ^1^, Fengxia Wang ^1^* and Lining Sun ^1^

^1^ J Jiangsu Provincial Key Laboratory of Advanced Robotics, School of Mechanical and Electric Engineering, Soochow University, Suzhou 215123, China.

^2^ Micro Nano System Research Center, Key Laboratory of Advanced Transducers and Intelligent Control System of Ministry of Education and Shanxi Province & College of Information Engineering, Taiyuan University of Technology, Taiyuan 030024, China

^3^ Suzhou Institute of Nano-tech and Nano-bionics, Chinese Academy of Sciences, Suzhou 215123.

The images were the position of the pulse, the simulation of iongel, the flow charts of sensor preparation and the acquisition system. The table showed the parameters and physiological significance of the pulse. Finally, the interpretation of cubic spline interpolation was given.


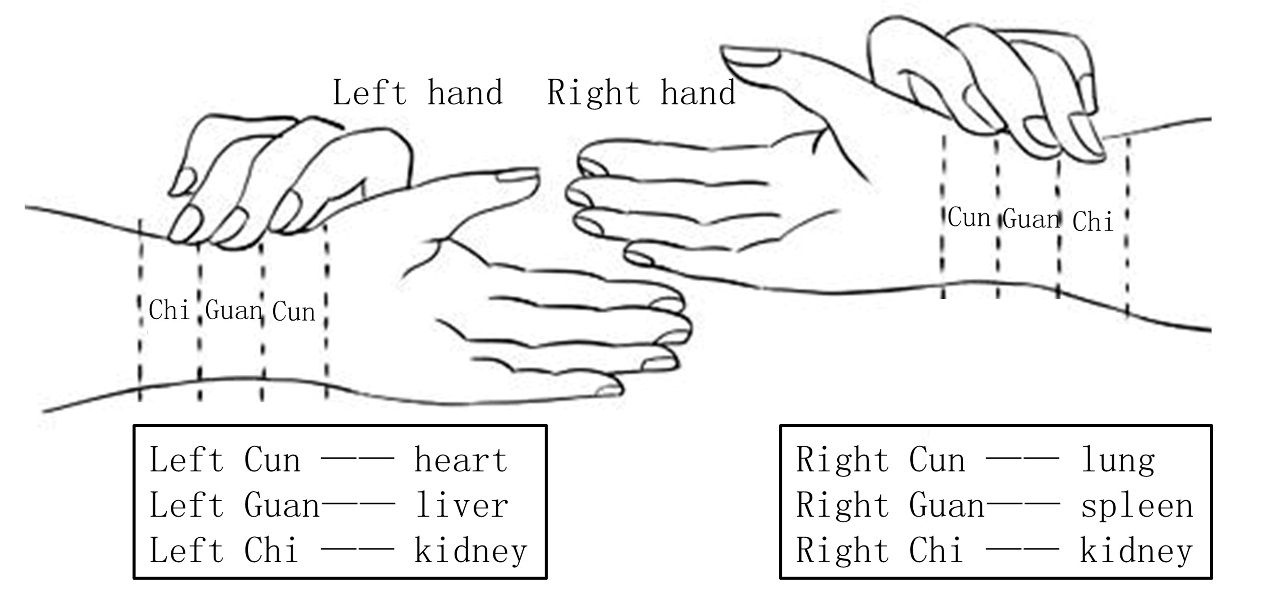


**Fig. S1** The three pulse positions on human wrist which could reflect the health status of corresponding human organs.


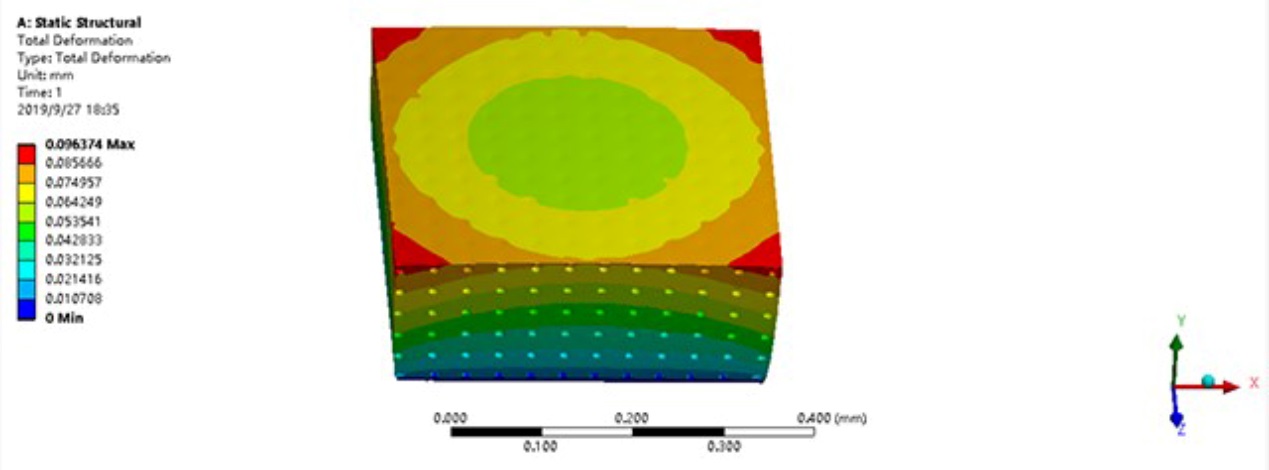


**Fig. S2** The simulation analysis of the ionogels loading 10KPa.


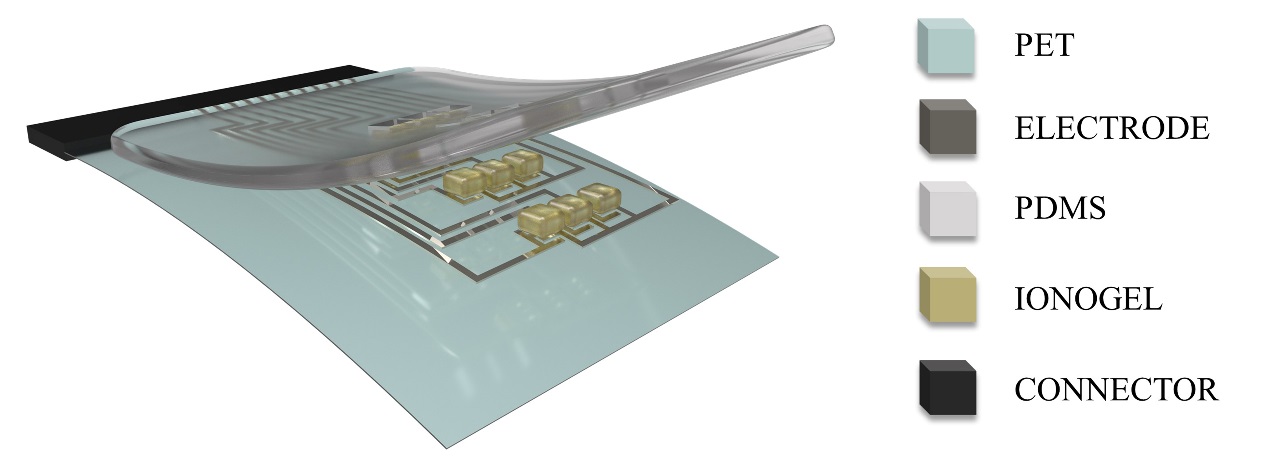


**Fig. S3** The flow chart of preparation of Ionogel based pulse sensor array


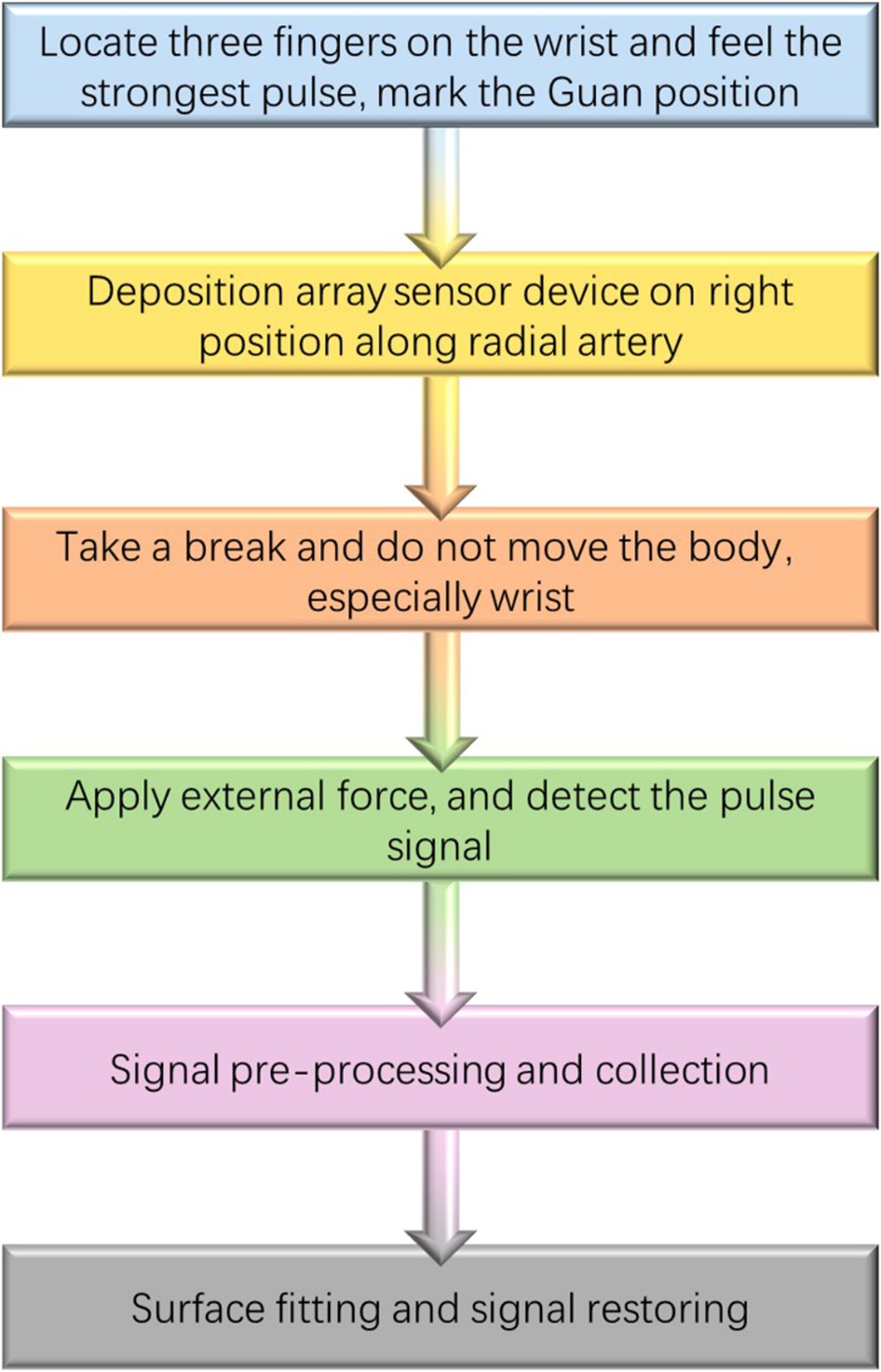


**Fig. S4** The signal process procedure of this pulse signal acquisition system.

**Table. S1** Common pulse and its corresponding physiological meaning


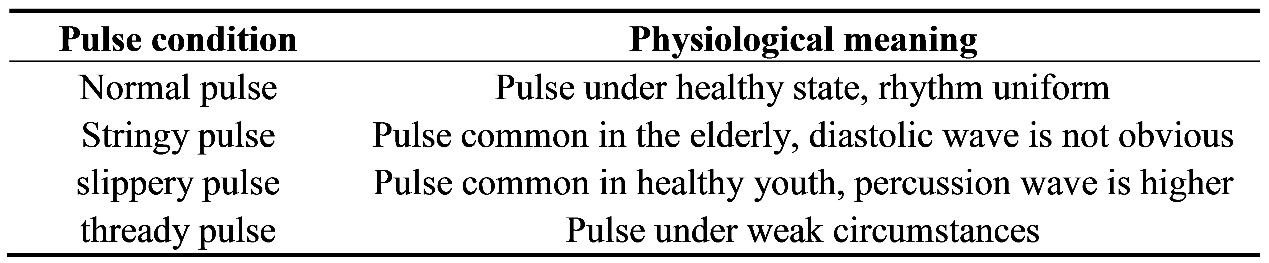


**Table. S2** Commonly used pulse time domain parameters and their corresponding physiological significance


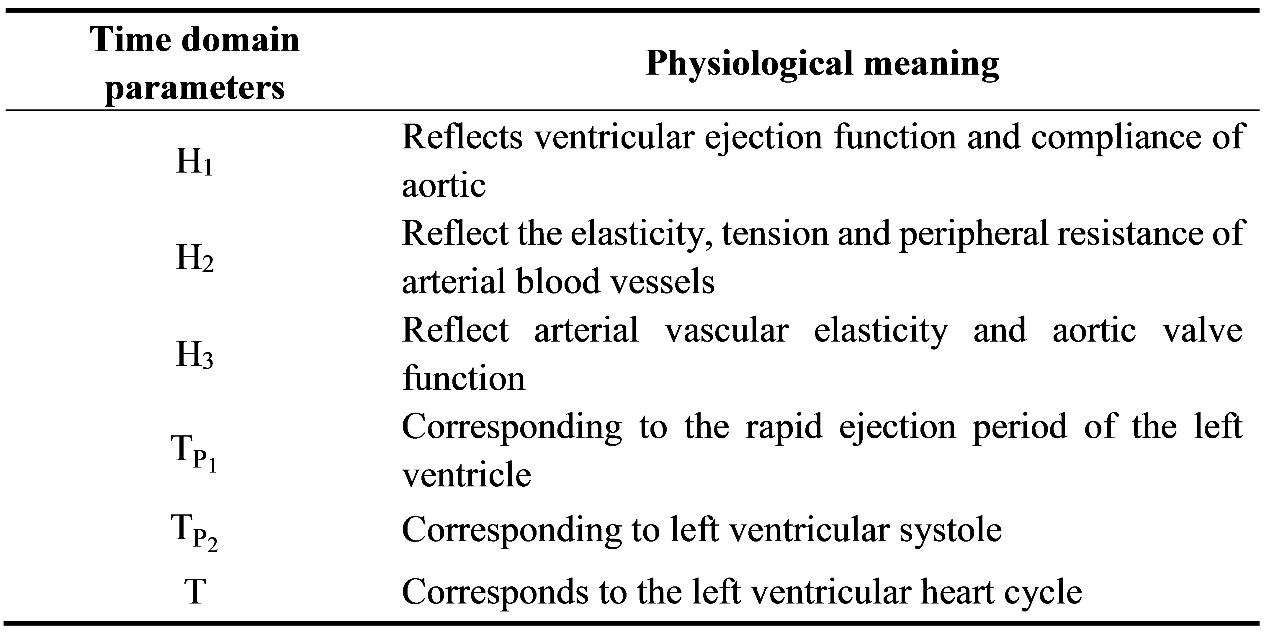


**Table. S3** Commonly used pulse wave construction parameters and their corresponding physiological significance


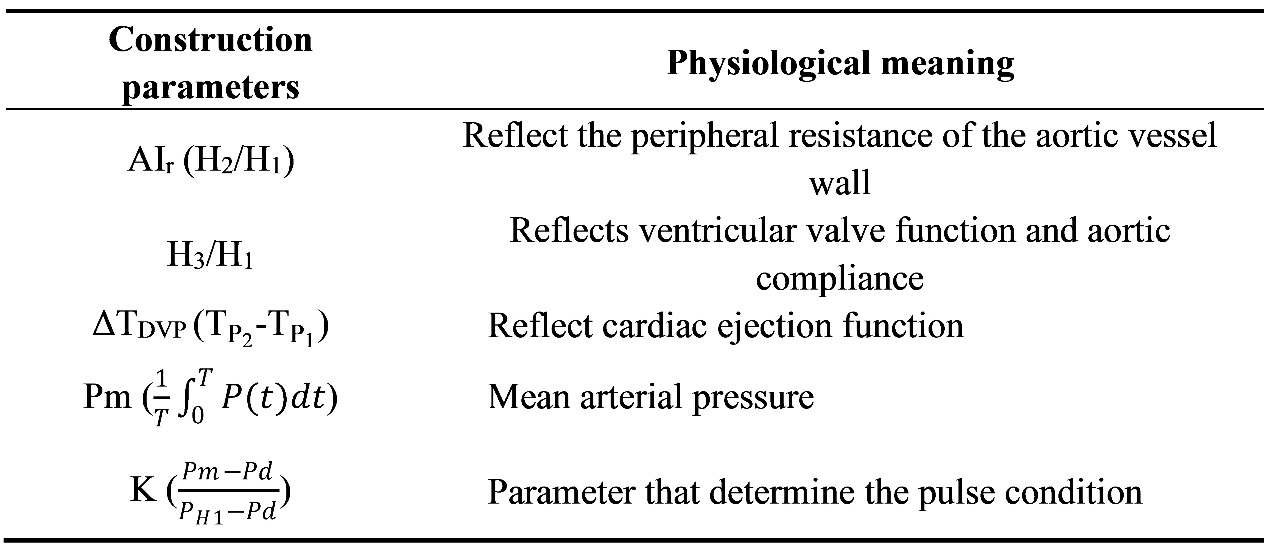


**Table. S4.**Comparison of average pulse parameters taken from different individuals


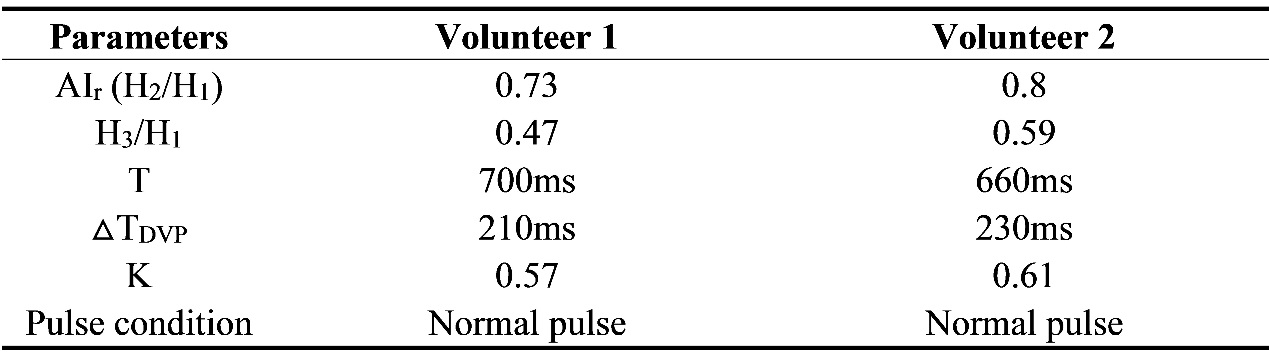


**Table. S5** Comparison of average pulse parameters under different physiological conditions


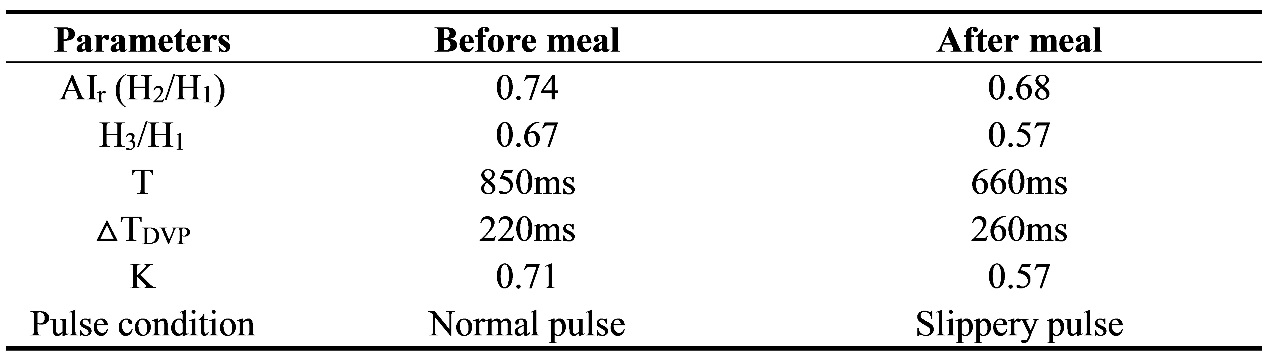


**Cubic spline interpolation**

Cubic spline interpolation is abbreviated to Spline interpolation, which is a process of obtaining curve function group by solving three moment equations through a smooth curve of a series of shape points. Its definition is: function S(x) ∈C^2^[a, b], and S(x) is a cubic polynomial on every interval [x_j_,x_j+1_], where a = x_0_ < x_1_ <... < x_n_= b is given nodes, then S(x) is called a cubic spline function on nodes x_0,_ x_1_…x_n_. If the value of the function Y_j_ = f (x_j_) (j =0, 1,…n) is given on the node x_j_ and S (x_j_) = y_j_ ( j =0, 1,…n)holds, then S(x) is called a cubic spline interpolation function.

In the actual calculation, it is necessary to introduce boundary conditions to complete the calculation. The boundary usually has a natural boundary (the second derivative of the boundary point is 0), a clamped boundary (the derivative of the boundary point is given), and a non-kink boundary (making the third-order derivative of the two endpoints equal to the third-order derivative of the adjacent points of the two endpoints). The definition of non-kink boundary is not specified in the general calculation method book, the numerical software such as Matlab takes the non-kink boundary condition as the default boundary condition.

The boundary condition refers to the variation of the variable or its derivative on the boundary of the solution region with time and place. The boundary condition is the premise that the governing equation has a definite solution, and it is necessary to give the boundary condition for any problem. The treatment of boundary conditions directly affects the accuracy of the calculation results. If there is a definite solution to the differential equation, the conditions must be introduced, and these additional conditions are called definite solution conditions.
